# Antibiotic Susceptibility Pattern of Bacterial Isolates in the Intensive Care Unit of a Tertiary Care Center in South India

**DOI:** 10.7759/cureus.113737

**Published:** 2026-07-31

**Authors:** Suraj K P, Prejeesh Balan, Praveen G S, Fairoz C P

**Affiliations:** 1 Pulmonary Medicine, Government Medical College, Kozhikode, Kozhikode, IND; 2 Pulmonary Medicine, Government TD Medical College, Alappuzha, Alappuzha, IND; 3 Microbiology, Government Medical College, Kozhikode, Kozhikode, IND

**Keywords:** antibiotics, culture, icu, resistance, sensitivity

## Abstract

Background and aims: There is an increase in the prevalence of antibiotic resistance noted in our intensive care units (ICU). The objective of this study is to evaluate the antibiotic susceptibility and resistance pattern in an ICU of a tertiary care hospital in India.

Materials and methods: This was a hospital-based prospective observational study done in a respiratory ICU of a tertiary care hospital in Kerala in South India. The study population included all subjects aged ≥ 18 years who were admitted to the ICU and required antibiotic treatment. Details of the culture and sensitivity pattern of the clinical isolates from sputum, blood, urine, endotracheal tube (ET) aspirate, and bronchoscopy specimens of the patients admitted to the ICU and requiring antibiotics were noted. Only the first isolate per patient was included while preparing the antibiogram.

Results: Out of the 154 patients enrolled, chronic obstructive pulmonary disease (COPD) was the most common diagnosis seen in 52 patients. The sputum culture positivity rate was 42%. Blood and urine culture positivity rates were 4% and 5%, respectively. ET aspirate had a positive culture report in 67% of the cases. Most of the organisms isolated were Gram-negative isolates such as *Klebsiella,* *Escherichia coli*, *Pseudomonas*, and *Acinetobacter*. *Klebsiella* was sensitive to colistin, tigecycline in 70%, meropenem in 66%, piperacillin-tazobactam in 60%, and cefoperazone-sulbactam in 58% of the isolates. *Escherichia coli* was sensitive to tigecycline (75%), colistin (70%), meropenem (68%), minocycline (65%), and piperacillin-tazobactam (64%). *Pseudomonas aeruginosa *was sensitive to colistin (74%), meropenem (71%), piperacillin-tazobactam (68%), and cefoperazone-sulbactam (64%). *Acinetobacter baumannii* was sensitive to tigecycline in 62%, meropenem in 54%, and piperacillin-tazobactam in 50%.

Conclusion: Gram-negative-resistant infections are increasing in our ICUs, leading to increased morbidity and mortality. Hence, timely antibiogram and antibiotic stewardship programs have to be implemented in our hospitals.

## Introduction

Antibiotics have played a major role in transforming modern medicine. The emergence of resistance and the lack of availability of new and effective antibiotics pose a grave threat to mankind, especially in developing countries like India [1]. According to the World Health Organization (WHO), antimicrobial resistance (AMR) is one of the 10 top global health threats. It undermines the effectiveness of various treatment programs and places a large number of people at risk of untreatable infections. According to the WHO report, in 2023, approximately one in six bacterial infections were resistant to antibiotics worldwide. Between 2018 and 2023, resistance to various essential antibiotics increased by 5-15% annually. AMR disproportionately affects low- and middle-income countries, with the highest rates being reported from Southeast Asia and the Eastern Mediterranean region [2].

Patients admitted to the intensive care unit (ICU) have an increased risk of developing healthcare-associated infections. Extremely vulnerable population groups with reduced host defense mechanisms, multiple procedures, and use of invasive devices like endotracheal intubation, central venous catheterization, mechanical ventilation (MV), and urinary catheterization, which distort the anatomical integrity and protective barriers of patients, are proposed reasons [3-5]. The majority of the nosocomial infections are caused by the ESKAPE pathogens, which include *Enterococcus faecium*, *Staphylococcus aureus*, *Klebsiella pneumoniae*, *Acinetobacter baumannii*, *Pseudomonas aeruginosa*, and *Enterobacter* species [6].

Both primary (at the time of admission) and secondary (occurring during the stay in the ICU or ICU-acquired) infection rates are higher in ICUs [7,8].

The common causes of community-acquired pneumonia (CAP) requiring ICU admission include *Streptococcus pneumoniae*, gram-negative bacilli (including *Klebsiella *and *Haemophilus influenzae*), atypical organisms (*Mycoplasma pneumoniae*), and viruses [9]. *Pseudomonas aeruginosa* is an important pathogen that causes CAP in patients with structural lung disease [10]. Several studies show that gram-positive organisms causing infections are more common in the West compared to gram-negative bugs, which are more common in ICUs in Asia [11]. In patients with risk factors for aspiration, like extremes of age, altered sensorium, dysphagia, and head and neck malignancy, there is a chance of infection with anaerobic organisms. *S. pneumoniae* is usually sensitive to beta-lactams and macrolides. *Haemophilus influenzae* shows good sensitivity to beta-lactams with beta-lactamase inhibitors and also fluoroquinolones. There is an increasing prevalence of extended-spectrum β-lactamase (ESBL)-producing *Enterobacteriaceae* in Indian ICUs [12].

On admission to the ICU with pneumonia, antibiotics are initially empirically started and later modified as per the culture and sensitivity reports. The antibiogram is a periodic profile of the antimicrobial susceptibilities of various organisms isolated from patients attending a hospital. It helps in guiding empirical antimicrobial therapy selection [13,14]. The pathogen distribution and antimicrobial resistance pattern change drastically with time. Hence, it is essential to periodically investigate changes in microbiology and antibiotic susceptibility patterns, especially in the ICU.

There is limited availability of recent antibiogram data from respiratory ICUs in South India. In this study, we analyze the pattern of antibiotic sensitivity and resistance based on the results of various cultures of microbial specimens from admitted patients. The findings may help guide empirical antibiotic selection in similar tertiary care ICUs.

## Materials and methods

The primary objective of the study was to evaluate the antibiotic sensitivity and resistance pattern in an ICU setting of a tertiary care hospital. This was a hospital-based prospective observational study conducted in the Respiratory ICU of a tertiary care center in Kerala, South India. The study was conducted from 01 March 2023 to 31 December 2023. The study population included all subjects aged ≥18 years who were admitted to the ICU and required antibiotic treatment. Patients or their nearest relative (for patients with subnormal conscious levels) not giving informed written consent and those without any signs and symptoms of systemic infection and not requiring antibiotics were excluded from the study. All necessary clearances were obtained from the Institutional Ethics Committee of Government Medical College, Kozhikode (Ref. No. GMCKKD/RP2023/IEC/107).

The minimum sample size for this study was calculated using the formula n = 4pq/d^2^. The culture positivity rate (p) was estimated at 37% (0.37) based on a study conducted by Chakraborty et al. [6], with q (100 - p) representing a non-positivity rate of 63% (0.63). Allowing a precision error (d) of 8% (0.08), the calculation yielded a required sample size of 146. The sampling technique used was simple convenience sampling. All consecutive patients admitted to the ICU who satisfied the inclusion criteria were enrolled in the study. Informed written consent was obtained. Clinical details were taken using a semi-structured proforma.

All the necessary clinical samples were collected appropriately, following the standard protocol at the bedside, and were then transported to the microbiology laboratory of the Department of Microbiology of our institute as early as possible. The clinical specimens processed included blood, urine, sputum, endotracheal tube (ET) aspirate, bronchioalveolar lavage fluid, pleural fluid, and throat swabs in indicated patients.

Clinical specimens collected were inoculated onto standard blood, MacConkey, and chocolate agar media. The plates were incubated aerobically at 37°C for 24-48 hours to isolate potential bacterial pathogens. Significant colonies were subsequently identified using standard biochemical tests to establish the profile. An automated bacterial identification system, Vitek 2 Compact (bioMérieux, Marcy-l'Étoile, France), was used to confirm the identity when needed.

Antimicrobial susceptibility testing (AST) was done by the Kirby-Bauer disc diffusion method on Mueller-Hinton agar plates. The zones of inhibition were measured after 18-24 hours of incubation at 37°C and interpreted according to the latest Clinical and Laboratory Standards Institute (CLSI M100, 33rd Edition, 2023) guidelines. The automated VITEK 2 system with species-specific ID and AST cards was used.

Quality control for AST was run weekly using ATCC reference strains *Staphylococcus aureus* ATCC 25923, *Escherichia coli* ATCC 25922, and *Pseudomonas aeruginosa* ATCC 27853. Only the first unique bacterial isolate per species from each individual patient was included in the cumulative antibiogram, regardless of the specimen source or subsequent variations in AST profiles. Repeat or subsequent isolates of the same species from the same patient were excluded to prevent statistical bias. Distinct, different bacterial species isolated from the same patient were treated as independent isolates and included in the analysis.

Details of culture reports and drug sensitivity were noted. Data collected were entered into a Microsoft Excel (Microsoft Corporation, Redmond, Washington) sheet. Data were represented as percentages, frequencies, and descriptive statistics (mean and standard deviation). Analysis was done using IBM SPSS Statistics for Windows, Version 30 (Released 2024; IBM Corp., Armonk, New York). Figures were made using MS Excel.

Multidrug resistance (MDR) was defined as acquired nonsusceptibility to at least one agent in three or more antimicrobial categories.

## Results

Demographic and baseline clinical characteristics

The study cohort comprised 154 participants with a predominantly elderly and male distribution. The baseline demographic data and clinical histories are detailed below.

Age Distribution

The mean age of the study population was 64 ±12 years, spanning a wide range from a minimum of 14 years to a maximum of 94 years.

Gender

Males accounted for the vast majority of the cohort at 76.0% (n = 117).

Social Habits

Active smoking history was documented in 63.0% (n = 97) of patients, and 33.0% (n = 52) had a history of chronic alcohol consumption.

Comorbidities and risks

Diabetes mellitus was present in 28.0% (n = 44) of the subjects. Immunosuppressive drug therapy was noted in 8.0% (n = 13) of patients. Notably, 39.0% (n = 61) of the cohort reported a history of antibiotic usage within the preceding three months.

Patient diagnoses and ventilatory support requirements

**Figure 1:**
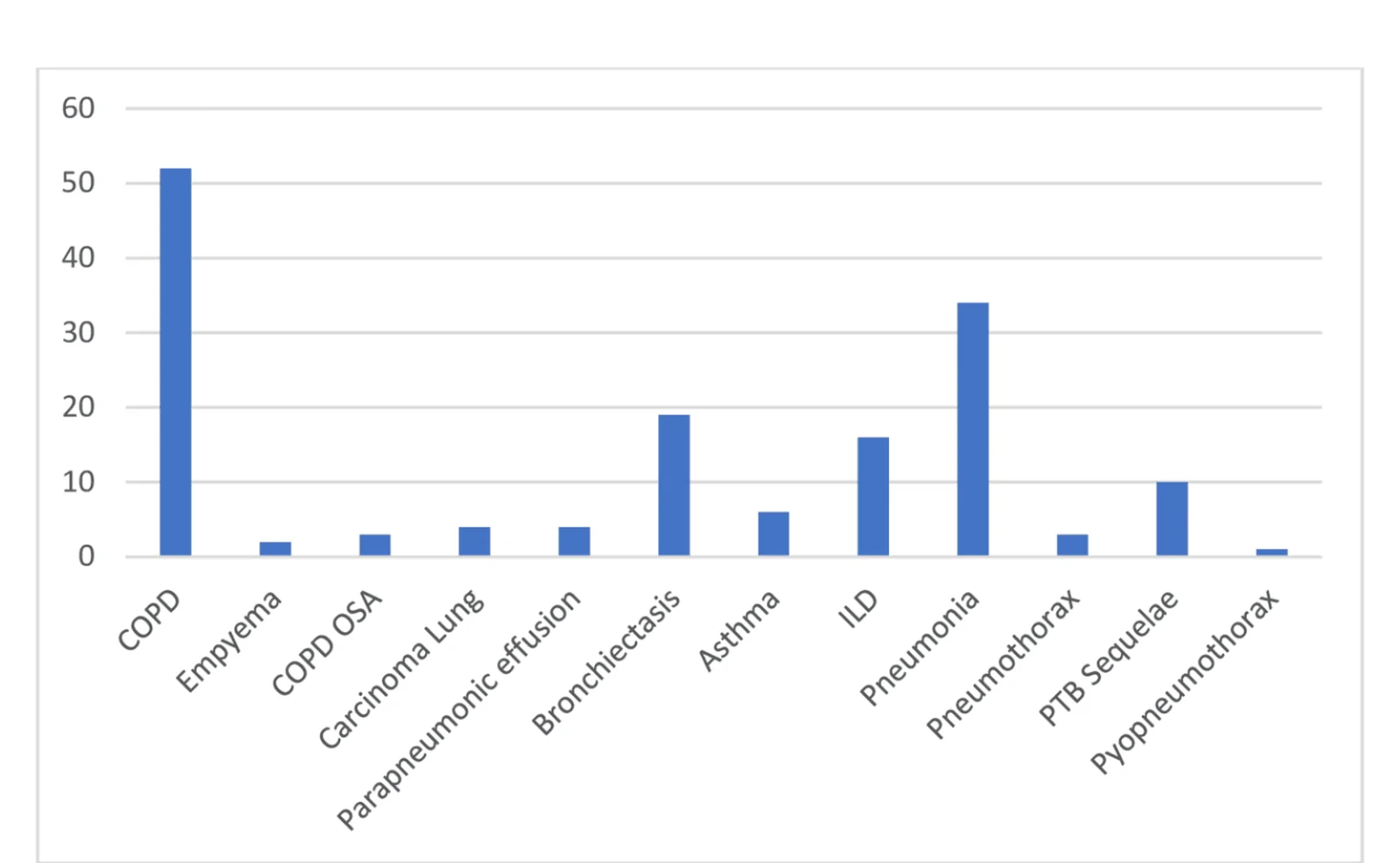
Diagnosis. Total Participants (n = 154)

Chronic obstructive pulmonary disease (COPD) was the most prevalent primary diagnosis, observed in 52 (33.7%) patients, followed by pneumonia in 34 (22.0%) patients (Figure 1).

Respiratory compromise was severe across the population: oxygen support was required by 153 patients (99.0%). Non-invasive ventilatory support (NIV) was administered to 88.0% (n = 136) of patients during their ICU stay, while 37.0% (n = 57) required invasive mechanical ventilation.

General microbiological profile by culture site

*Sputum Cultures*

**Figure 2:**
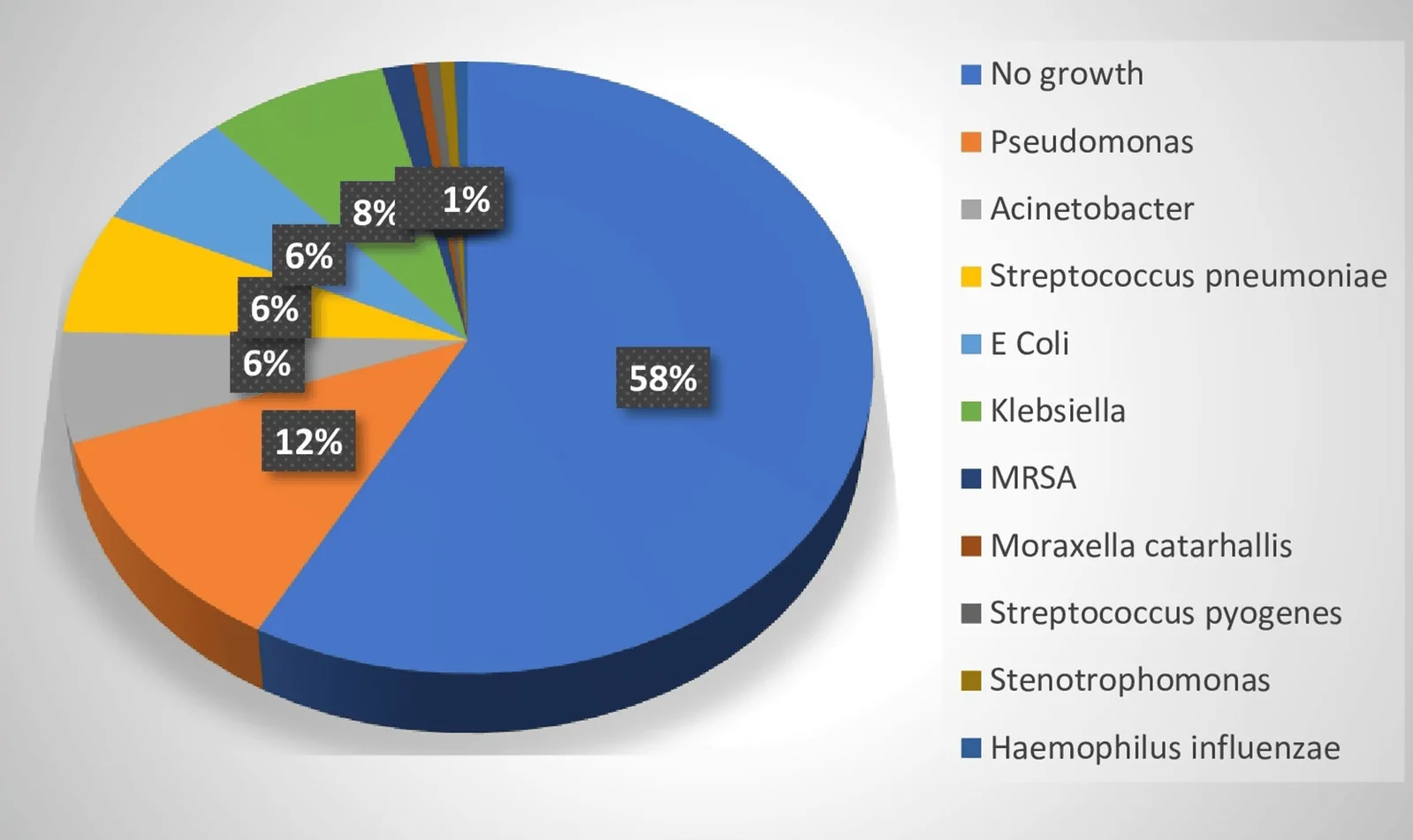
Sputum Culture Positivity Rate. No growth 58% (n = 89)

Sputum culture analysis revealed no bacterial growth in the majority of cases, accounting for 58% (n = 89) of the sample population (Figure 2). Among the positive sputum cultures, *Pseudomonas* was the most frequently isolated pathogen, found in 12% (n = 18) of cases. This was followed by *Klebsiella* in 8% (n = 12). *Acinetobacter*, *Escherichia coli*, and *Streptococcus pneumoniae* were each isolated in 6% (n = 10) of the sputum samples.

*Blood and Urine Cultures*

**Figure 3:**
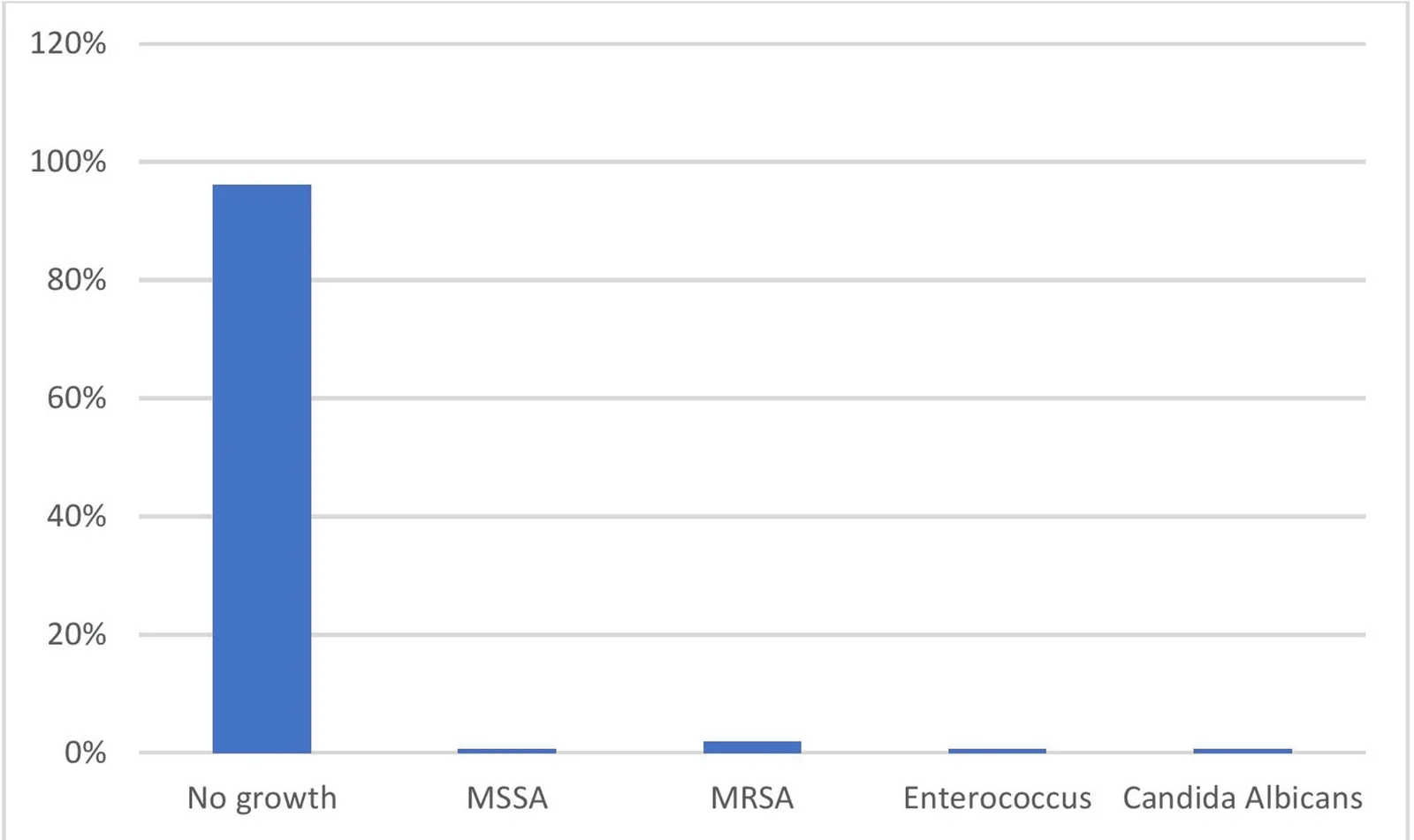
Blood Culture Positivity Rate. No Growth 96% (n = 148)

Blood cultures demonstrated an extremely low yield of pathogen isolation, with 96% (n = 148) showing no bacterial growth (Figure 3). The most common organism recovered from blood cultures was methicillin-resistant *Staphylococcus aureus* (MRSA), which was detected in three patients (1.9%). Similarly, urine cultures remained negative for 95% (n = 147) of the samples, with *E. coli* isolated as the predominant pathogen in the remaining five samples (3%).

*ET Aspirate Cultures*

**Figure 4:**
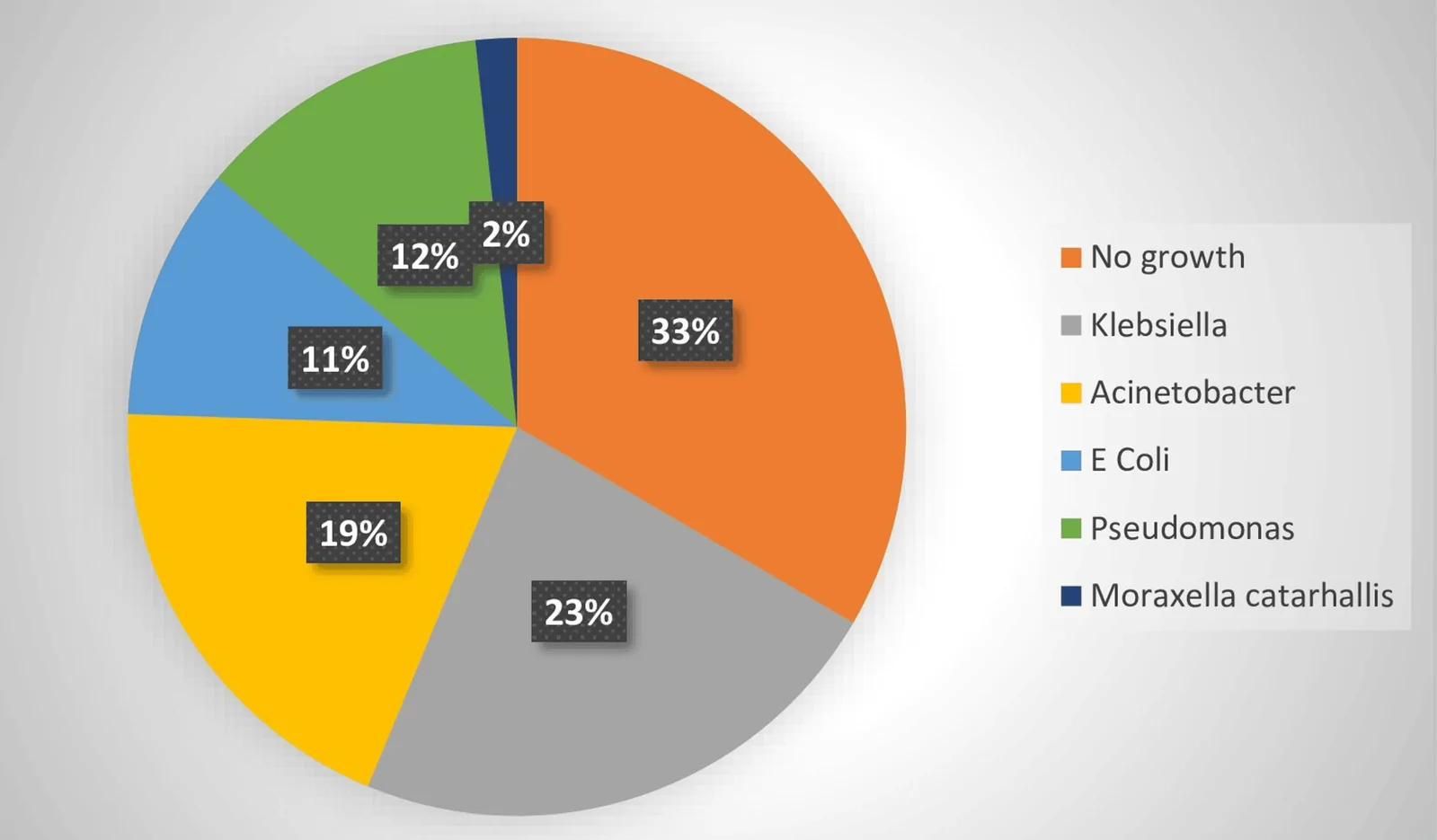
Endotracheal Tube Aspirate Culture Percentage. Positive Culture 67% (n = 38)

ET aspirate samples were collected and submitted for culture from 57 patients. The culture positivity rate was high at 67% (n = 38) (Figure 4). The leading pathogens recovered from these specimens were *Klebsiella* at 23% (n = 13), followed closely by *Acinetobacter* at 19% (n = 11) and *Pseudomonas* at 12% (n = 7).

*Other Samples*

Pleural fluid aspirates were evaluated in 13 subjects. Sterile samples with no growth were observed in 38% (n = 5) of these patients, while *Pseudomonas* was isolated in four cases (30%). Bronchoalveolar lavage (BAL) fluid was cultured for 20 patients, yielding a high positive culture rate of 70% (n = 14). The most common isolates from BAL fluid included *Acinetobacter* (25%, n = 5), *Pseudomonas* (25%, n = 5), and *E. coli* (20%, n = 4). Additionally, throat swabs collected from two patients (50% of those swabbed) tested positive for the H1N1 influenza virus.

Pathogen stratification by primary respiratory diagnosis

*COPD*

**Figure 5:**
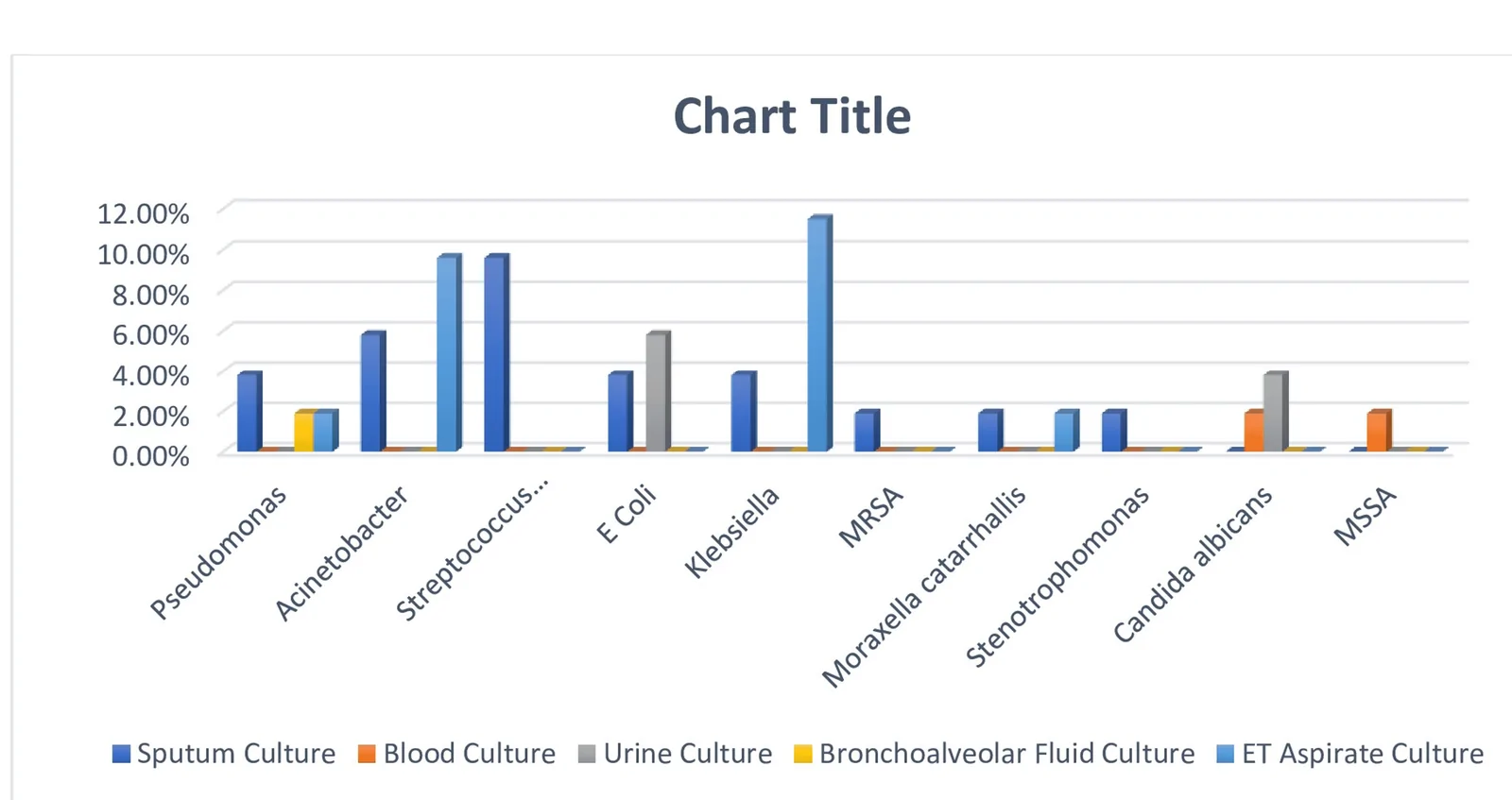
Chronic Obstructive Pulmonary Disease (COPD) (n = 52)

In the 52 COPD patients, sputum cultures showed a 32.7% (n = 17) positivity rate, with *S. pneumoniae* being the most common organism (9.6%, n = 5). Conversely, ET aspirate cultures displayed a striking 80.8% (n = 13) positivity rate, primarily yielding *Klebsiella* spp. (11.5%, n = 6) and *Acinetobacter* spp. (9.6%, n = 5) (Figure 5).

*Pneumonia*

**Figure 6:**
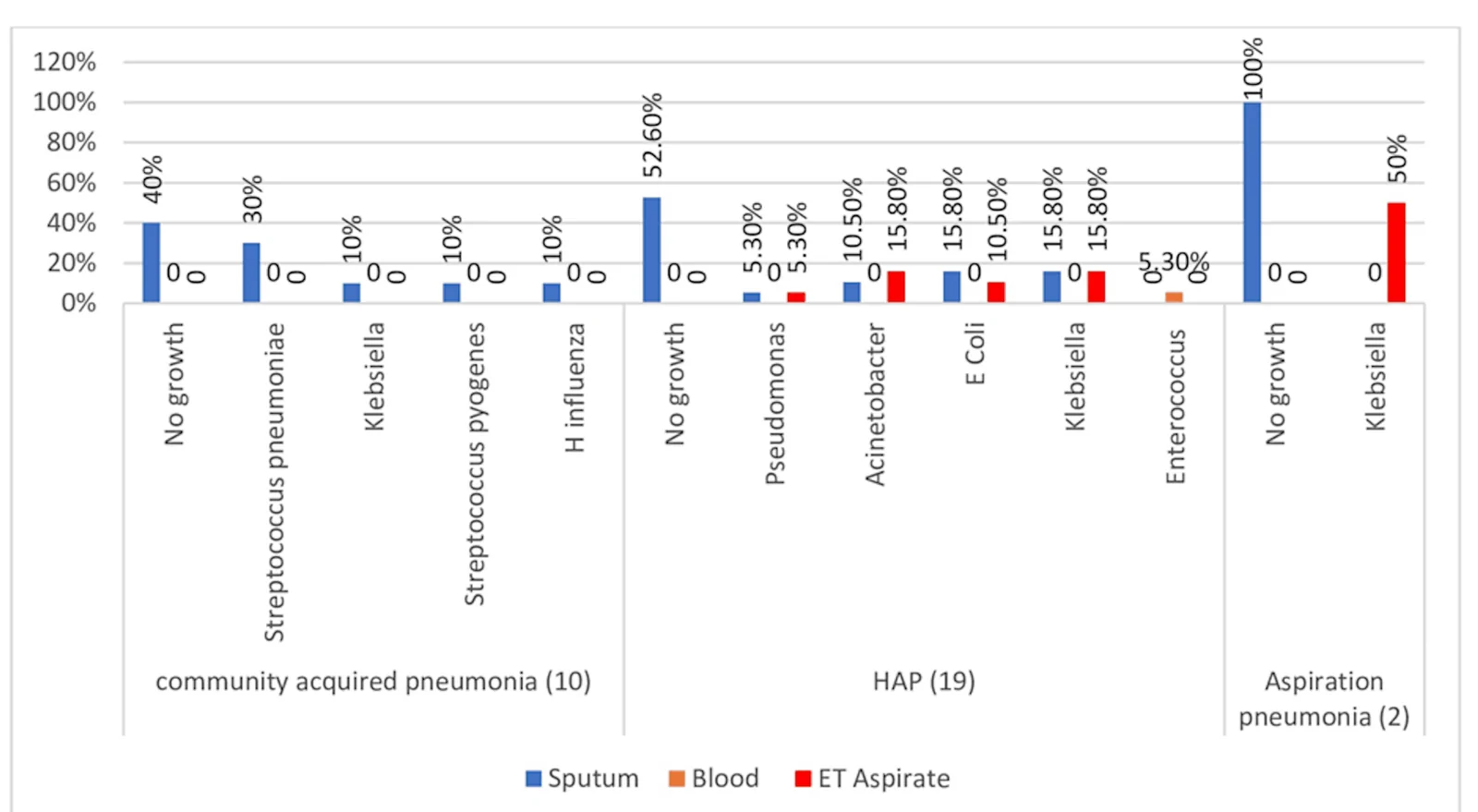
Pneumonia (n = 34) CAP = 10, HAP = 19, aspiration pneumonia = 2, viral pneumonia = 2, *Pneumocystis jirovecii* pneumonia = 1 CAP: community-acquired pneumonia; HAP: hospital-acquired pneumonia

The pneumonia subgroup (n = 34) comprised hospital-acquired pneumonia (HAP; n = 19), CAP (n = 10), aspiration pneumonia (n = 2), viral pneumonia (n = 2), and *Pneumocystis jirovecii* pneumonia (n = 1) (Figure 6).

CAP: Sputum cultures yielded no growth in 40% (n = 4) of patients. *Streptococcus pneumoniae* was the most common isolated organism, detected in 30% (n = 3) of cases.

HAP: Sputum cultures showed no growth in 52.6% (n = 10) of patients. The isolated pathogens from sputum included *E. coli* (15.8%, n = 3), *Klebsiella* (15.8%, n = 3), *Acinetobacter* (10.5%, n = 2), and *Pseudomonas* (5.3%, n = 1). ET aspirate cultures for HAP patients also frequently grew *Acinetobacter* (15.8%, n = 3) and *Klebsiella* (15.8%, n = 3).

Aspiration pneumonia: Both patients presented with negative sputum cultures, though one patient (50%) demonstrated ET aspirate culture positivity for *Klebsiella*.

*Bronchiectasis*

**Figure 7:**
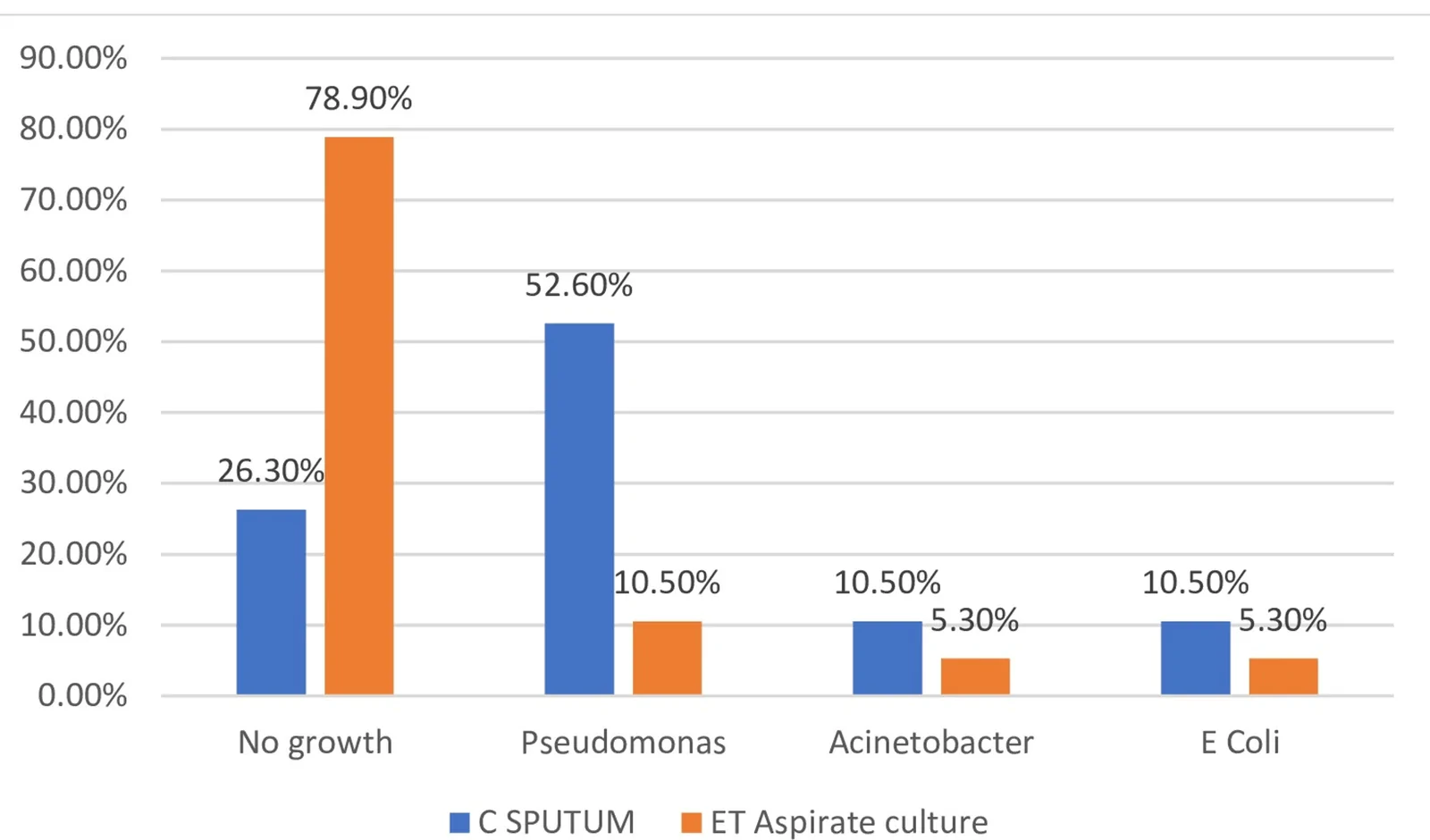
Bronchiectasis (n = 19)

In the bronchiectasis cohort (n = 19), *Pseudomonas* was identified as the predominant offending pathogen. It was the most common isolate across both sample types, recovered from 52.6% (n = 10) of sputum cultures and 10.5% (n = 2) of ET aspirate cultures (Figure 7).

Antimicrobial sensitivity and resistance pattern

To address the primary objective of determining ICU antibiotic susceptibility profiles, standard sensitivity testing was conducted on the major isolates.

*Streptococcus pneumoniae (n = 10)*

**Figure 8:**
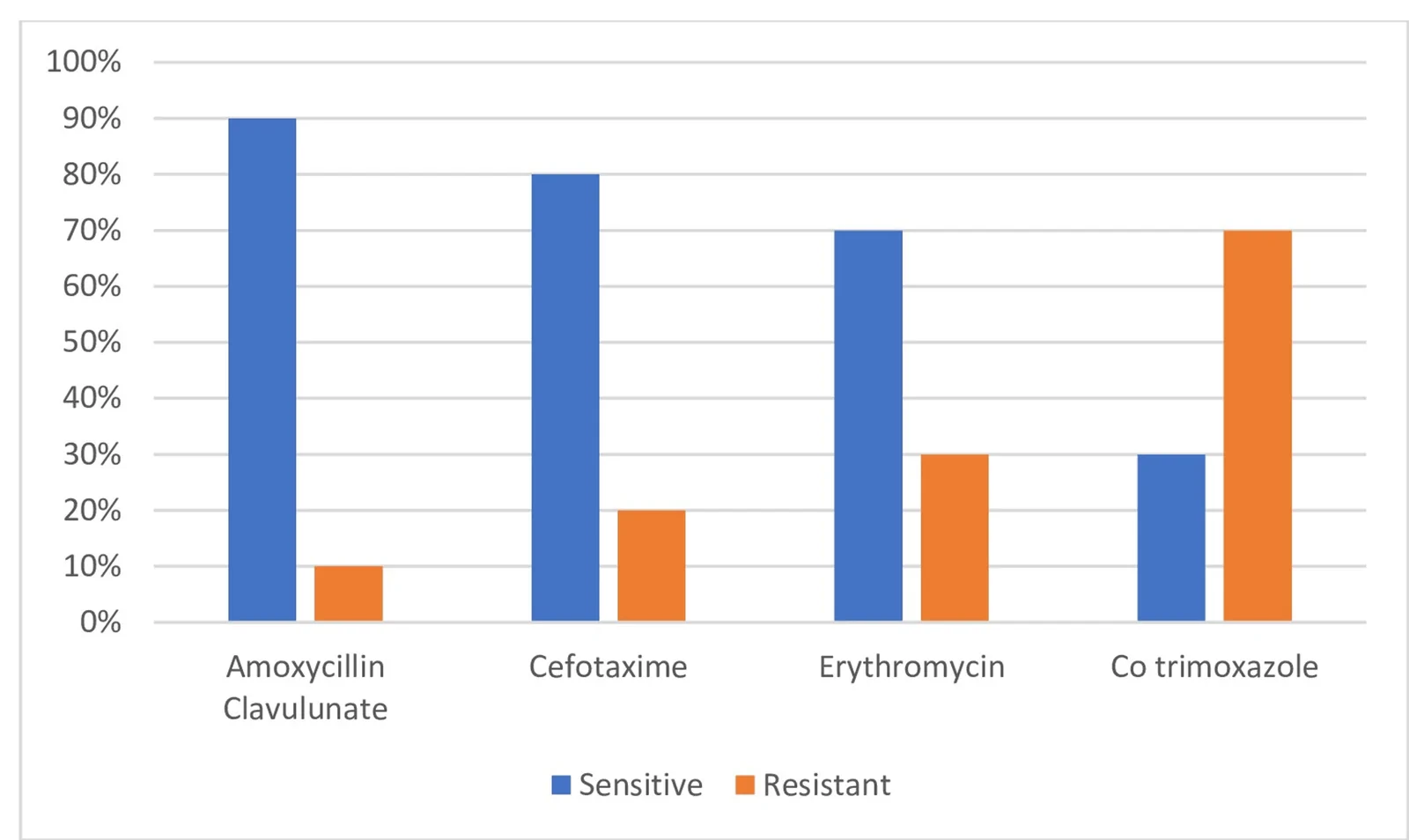
Streptococcus pneumoniae (n = 10) Sensitivity and Resistance Pattern

Isolates of *Streptococcus pneumoniae* demonstrated high susceptibility to standard first-line therapies. Amoxicillin-clavulanate was active against 90% (n = 9) of isolates, and cefotaxime showed 80% (n = 8) sensitivity. However, a significant resistance rate of 70% (n = 7) was observed against co-trimoxazole (Figure 8).

*Pseudomonas aeruginosa (n = 21)*

**Figure 9:**
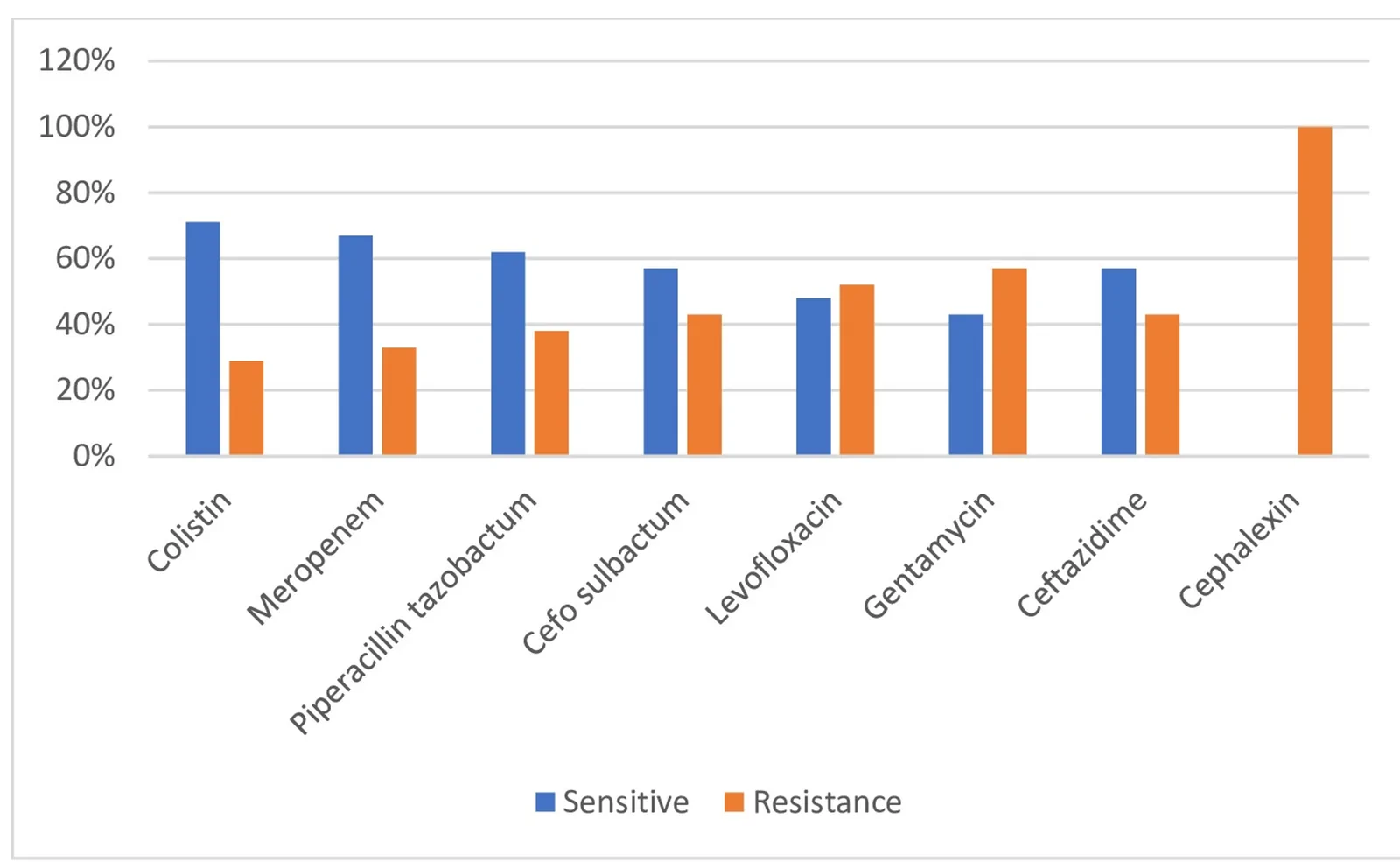
Pseudomonas aeruginosa (n = 21) Sensitivity and Resistance Pattern

The 21 *Pseudomonas aeruginosa* isolates showed the highest sensitivity to colistin at 71% (n = 15), followed by meropenem at 67% (n = 14), piperacillin-tazobactam at 62% (n = 13), and cefoperazone-sulbactam at 57% (n = 12). Complete resistance (100%) was observed against cephalexin. Resistance to gentamycin and ceftazidime was recorded at 57% (n = 12) and 43% (n = 9), respectively (Figure 9).

*Acinetobacter baumannii (n = 20)*

**Figure 10:**
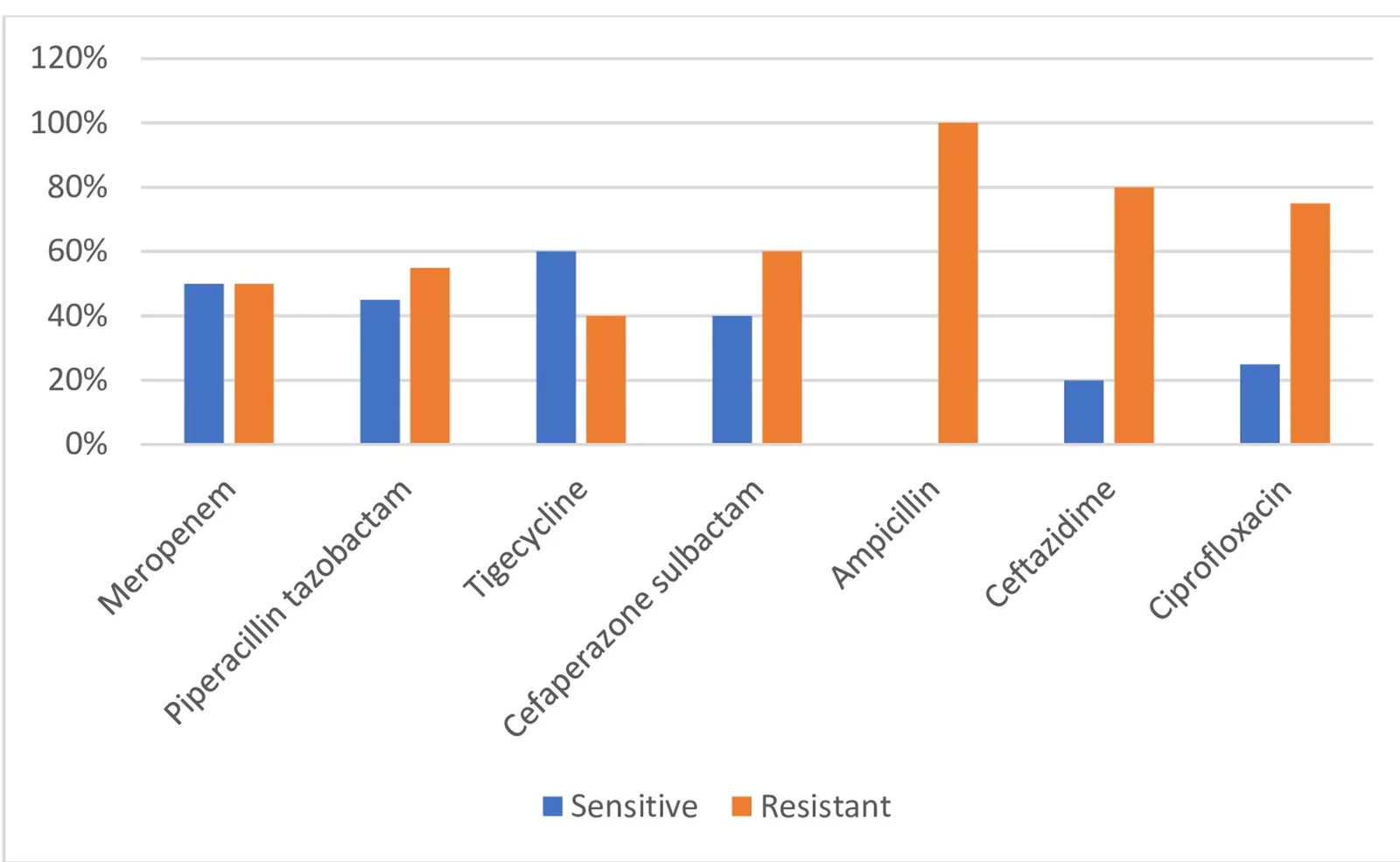
Acinetobacter baumannii (n = 20) Sensitivity and Resistance Pattern

Among the 20 *Acinetobacter baumannii* isolates, the most effective antimicrobial agent was tigecycline with a 60% (n = 12) sensitivity rate, followed by meropenem at 50% (n = 10) and piperacillin-tazobactam at 45% (n = 9). Universal resistance (100%) was documented against ampicillin. High resistance rates were also observed for ceftazidime (80%, n = 16) and ciprofloxacin (75%, n = 15) (Figure 10).

*Escherichia coli (n = 18)*

**Figure 11:**
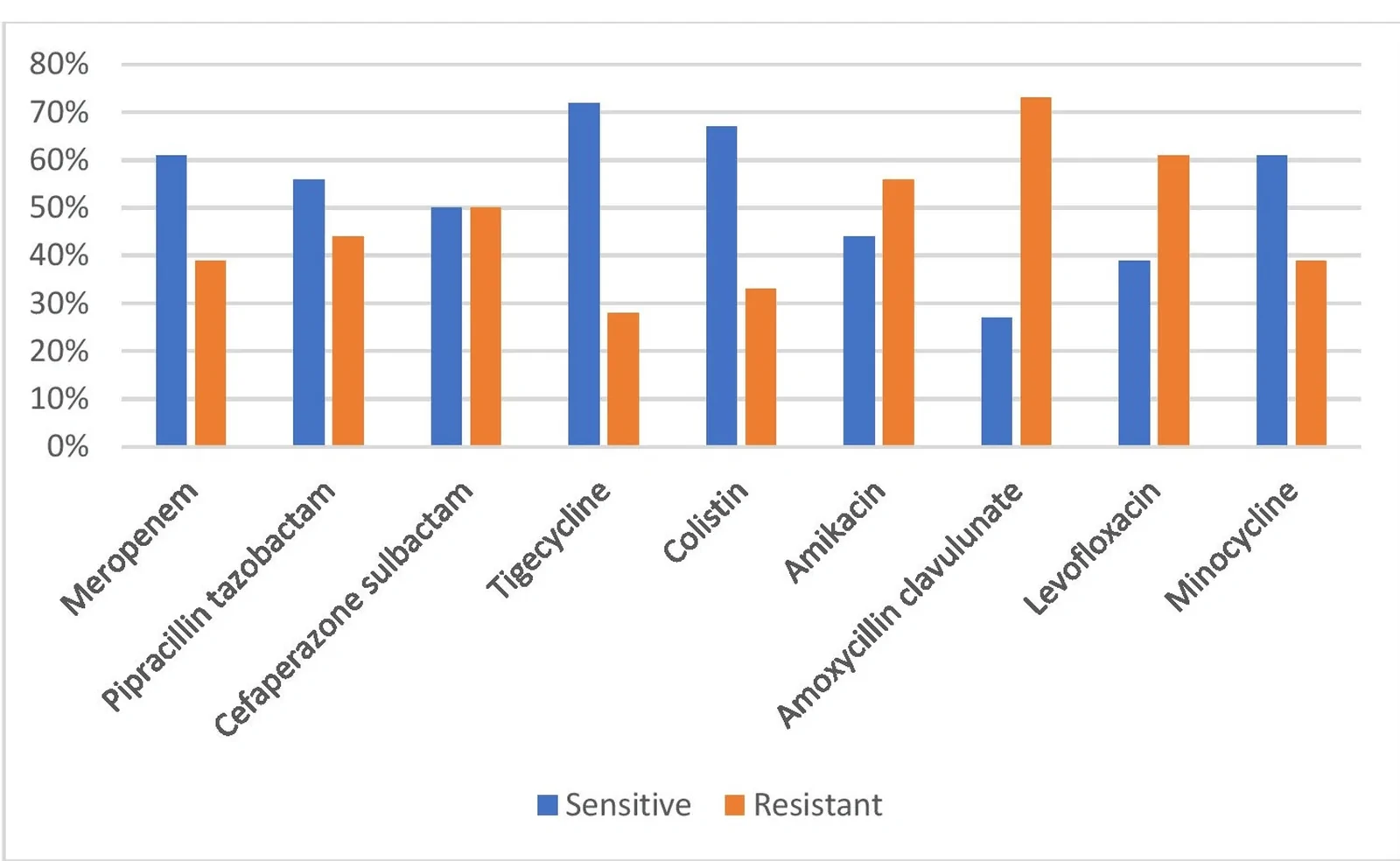
Escherichia coli (n = 18) Sensitivity and Resistance Pattern

The 18 *Escherichia coli* isolates displayed favorable susceptibility to tigecycline (72%, n = 13) and colistin (67%, n = 12). Susceptibility to both meropenem and minocycline stood at 61% (n = 11), while sensitivity to piperacillin-tazobactam was 56% (n = 10). A high resistance rate of 73% (n = 13) was noted against amoxicillin-clavulanate (Figure 11).

*Klebsiella spp. (n = 24)*

**Figure 12:**
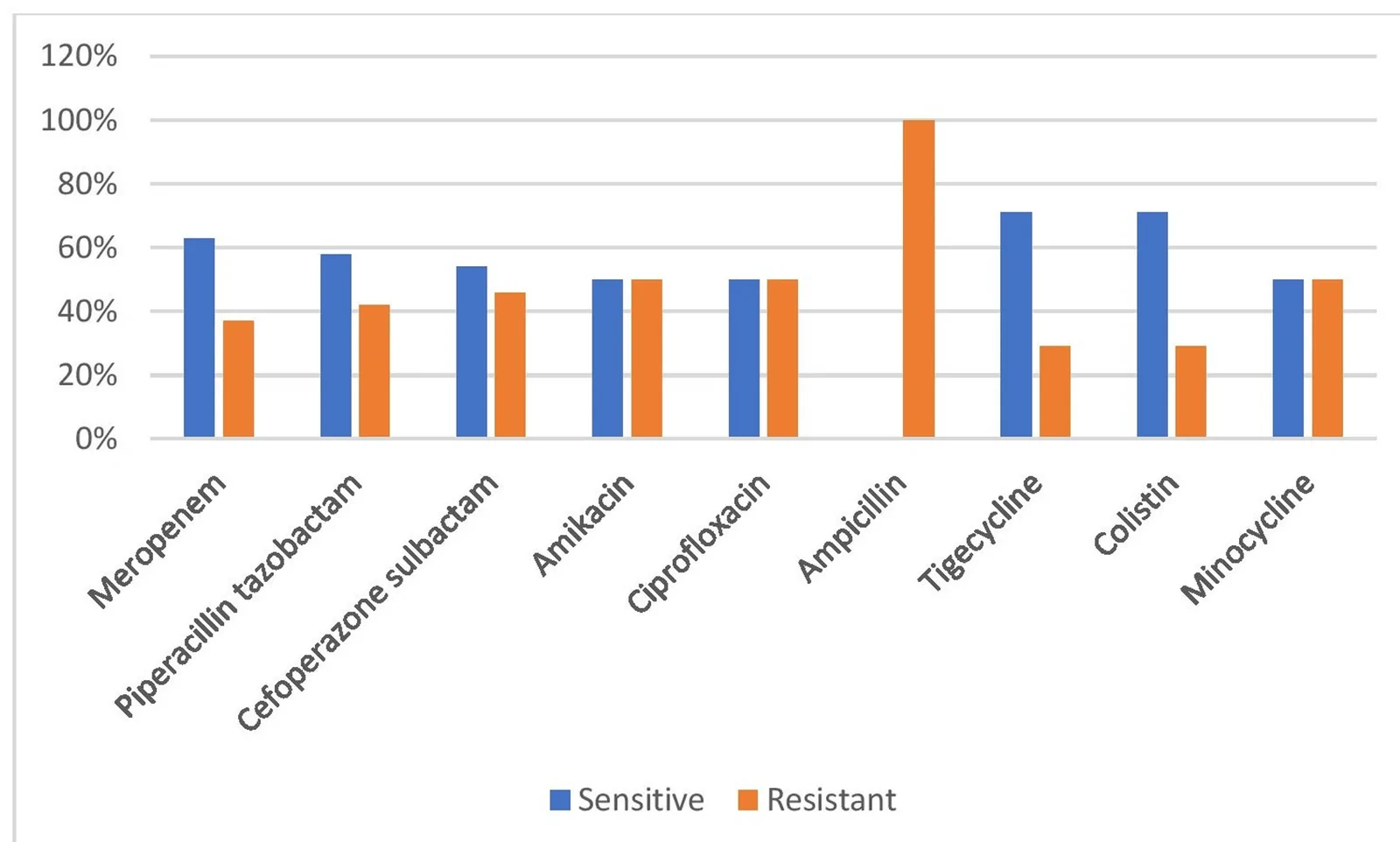
Klebsiella (n = 24) Sensitivity and Resistance Pattern

For the 24 *Klebsiella* isolates, the highest rates of sensitivity were shared by colistin and tigecycline at 71% (n = 17) each. Susceptibility to other agents included meropenem at 63% (n = 15), piperacillin-tazobactam at 58% (n = 14), and cefoperazone-sulbactam at 54% (n = 13). Universal resistance (100%) was observed against ampicillin, and 50% (n = 12) of the isolates were resistant to ciprofloxacin (Figure 12).

## Discussion

AMR has emerged as a major global threat in critical care medicine. In the ICU, drug resistance directly complicates patient management, worsens clinical prognosis, compromises survival, prolongs hospital stays, and exponentially inflates the financial burden of treatment. This study evaluated the local microbiological footprint and susceptibility patterns to optimize empirical therapy and improve patient clinical outcomes.

The demographic characteristics of this study indicate a vulnerable population. The mean age of 64 years combined with high rates of smoking (63.0%) and alcohol use (33.0%) underscores a high baseline risk for advanced structural lung diseases, which correlates with COPD and pneumonia dominating the clinical diagnoses. Furthermore, nearly 40% of the patients had a recorded history of recent antibiotic exposure. This prior exposure is a well-established driver for selecting multidrug-resistant strains prior to or during ICU admission.

Diagnostic yield and culture positivity rates

The diagnostic yield in this study varied significantly depending on the anatomical site and sample type. The culture positivity rate reached 42.0% in sputum samples, 67.0% in ET aspirate samples, and 70.0% in BAL fluid. Conversely, systemic and urinary yields were remarkably low, with blood and urine cultures demonstrating positivity rates of only 4.0% and 5.0%, respectively.

These diagnostic yields align broadly with established critical care literature, though variations exist based on geographic context and patient populations. Savanur et al. reported a substantially higher overall culture positivity rate of 76.0% [1], which may reflect a cohort with higher systemic baseline infections or different clinical sampling thresholds. Conversely, our findings sit comfortably alongside the work of Chakravarthi et al., who reported a comparable overall culture positivity rate of 46.4% [15]. The high yield observed in ET aspirates and BAL fluid in this study underscores the critical utility of lower respiratory tract sampling in intubated patients to identify true pathogenic architecture versus superficial upper airway colonization.

Pathogen distribution by sample type and global trends

The overall microbiological profile in our ICU was overwhelmingly dominated by GNB, particularly within the respiratory tract specimens. Sputum samples grew *Pseudomonas* spp. (12.0%), *Klebsiella* spp. (8.0%), *Acinetobacter* spp. (6.0%), and *Escherichia coli* (6.0%), with *Streptococcus pneumoniae* (6.0%) standing out as the only major Gram-positive respiratory isolate. ET aspirate specimens displayed an identical preference for GNB, with higher specific rates for *Klebsiella* spp. (23.0%), *Acinetobacter* spp. (19.0%), and *Pseudomonas* spp. (12.0%). Meanwhile, *Staphylococcus aureus* (primarily MRSA) and *E. coli* remained the leading isolates from the few positive blood and urine cultures, respectively.

This massive predominance of Gram-negative pathogens mirrors structural epidemiological shifts documented across Asian critical care settings. In contrast to Western ICUs, where Gram-positive organisms like *Staphylococcus aureus* and *Enterococcus* spp. frequently lead nosocomial counts, Asian healthcare structures persistently battle high endemic rates of MDR *Enterobacteriaceae* and non-fermenting GNB [1,15], an observation heavily reinforced by our data.

Etiological stratification by respiratory disease

*COPD*

Among the 52 patients presenting with acute exacerbations of COPD, *S. pneumoniae* (9.6%) was the primary pathogen recovered from sputum, whereas *Klebsiella* spp. (11.5%) and *Acinetobacter* spp. (9.6%) led the yields in ET aspirate samples. Susheela et al. reported a significantly higher rate of *S. pneumoniae* isolation at 22.0% [16], and Sharma et al. also documented it as the leading singular organism at 13.0%. Crucially, however, Sharma et al. noted that when individual GNB species were pooled together, Gram-negative bacteria collectively formed the absolute predominant etiological block [17]. This matches our observations, suggesting that while classic pathogens drive early community-based exacerbations, severe cases requiring invasive instrumentation quickly shift toward nosocomial GNB.

CAP vs. HAP

Clear etiological boundaries were observed when stratifying the pneumonia subgroup (n = 34). CAP: *S. pneumoniae* was confirmed as the principal pathogen, identified in 30.0% of cases. This is virtually identical to the 30.5% rate reported by Para et al. [18], validating classic empirical CAP algorithms. HAP: The microbial flora shifted completely to nosocomial GNB, led by *E. coli* (15.8%), *Klebsiella* spp. (15.8%), *Acinetobacter* spp. (10.5%), and *Pseudomonas* spp. (5.3%). This finding agrees with Tharavichitkul et al., who identified *Acinetobacter baumannii* as a premier cause of HAP [19], and Mukhopadhyay et al., who highlighted *Pseudomonas* spp. as a principal driver of nosocomial lung infections [20].

*Bronchiectasis*

In our bronchiectasis cohort (n = 19), *Pseudomonas* spp. was highly prevalent, isolated in 52.6% of patients across sputum and ET aspirates. While Dhar et al. also identified *Pseudomonas aeruginosa* as the most frequent isolate in bronchiectasis, their documented rate was noticeably lower at 13.7% [21]. The exceptionally high concentration of *Pseudomonas* in our cohort likely reflects advanced structural parenchymal damage and a high frequency of repeat hospitalizations among patients requiring ICU-level care.

Antimicrobial susceptibility and resistance patterns

*Gram-Positive Pathogens: Streptococcus pneumoniae*

Our 10 *S. pneumoniae* isolates demonstrated highly encouraging susceptibility to standard beta-lactams, preserving a 90.0% sensitivity rate to amoxicillin-clavulanate and an 80.0% sensitivity rate to cefotaxime. However, a major resistance barrier was observed against co-trimoxazole (70.0%). This high resistance ceiling matches the baseline trends noted by Chawla et al., who reported peak resistance markers for co-trimoxazole and tetracycline [22], confirming that antifolate agents are no longer reliable empirical options for pneumococcal infections.

*Non-Fermenting GNB*

The resistance profiles of our non-fermenting GNB isolates reveal an urgent clinical situation.

*Pseudomonas aeruginosa* (n = 21): This pathogen showed the highest sensitivity to reserve and traditional anti-pseudomonal agents: colistin (71.0%), meropenem (67.0%), piperacillin-tazobactam (62.0%), and cefoperazone-sulbactam (57.0%). Universal resistance (100.0%) was observed for cephalexin, along with high resistance to gentamicin (57.0%) and ceftazidime (43.0%). Interestingly, Savanur et al. recorded significantly higher resistance rates across all anti-pseudomonal classes, including ceftazidime (76.4%), piperacillin-tazobactam (64.7%), and meropenem (41.1%) [1]. While our isolates were comparatively more manageable, the 33.0% carbapenem resistance rate remains a major clinical concern.

*Acinetobacter baumannii* (n = 20): This organism proved to be the most resistant pathogen in our unit. It maintained moderate sensitivity only to tigecycline (60.0%) and meropenem (50.0%), while showing extensive resistance to ampicillin (100.0%), ceftazidime (80.0%), and ciprofloxacin (75.0%). This profile mirrors the extreme resistance trends noted by Savanur et al., where cephalosporin resistance hit 96.0% and piperacillin-tazobactam resistance reached 84.0% [1]. Furthermore, Jaggi et al. documented a massive 93.2% carbapenem resistance rate in *Acinetobacter* strains [23]. Our 50.0% carbapenem resistance rate indicates that while we are below certain national averages, our therapeutic window for this pathogen is narrowing rapidly.

*Enterobacteriaceae (E. coli and Klebsiella spp.)*

The resistance markers among our *Enterobacteriaceae* isolates highlight a high prevalence of ESBL production and emerging carbapenem resistance.

*Escherichia coli* (n = 18): These isolates were sensitive to tigecycline (72.0%), colistin (67.0%), meropenem (61.0%), and minocycline (61.0%). However, *E. coli* exhibited a 73.0% resistance rate to amoxicillin-clavulanate. Banu et al. similarly reported high resistance to ampicillin (96.4%) and co-trimoxazole (74.2%), though all of their isolates retained complete imipenem sensitivity [24]. Savanur et al. also reported high resistance to third- and fourth-generation cephalosporins (62.5%) [1], mirroring the high rates of ESBL-driven aminopenicillin failure observed in our study.

*Klebsiella* spp. (n = 24): These strains showed shared peak sensitivities to colistin (71.0%) and tigecycline (71.0%), followed by meropenem (63.0%) and piperacillin-tazobactam (58.0%). Complete resistance (100.0%) was noted against ampicillin, and 50.0% were resistant to ciprofloxacin. In the comparative work by Savanur et al., *Klebsiella* exhibited 65.0% resistance to cephalosporins, 60.0% resistance to meropenem, and an emerging 20.0% resistance rate to colistin [1]. Sheth et al. similarly highlighted extensive cephalosporin resistance in *Klebsiella* [25]. Our findings confirm that carbapenem resistance in *Klebsiella* has established a strong presence in the ICU.

Clinical implications, resistance drivers, and antibiograms

The high resistance to second- and third-generation cephalosporins among our major GNB isolates (*Klebsiella*, *E. coli*, *Pseudomonas*, *Acinetobacter*) is a defining feature of this study. Historically, piperacillin-tazobactam and carbapenems have been used as the standard empiric choices for severely ill patients in Indian ICUs. However, we observed a high resistance rate to piperacillin-tazobactam, ranging from 38.0% to 55.0% across these species. Furthermore, the notable increase in carbapenem-resistant *Enterobacteriaceae* (CRE) over the last decade is clearly reflected in our data, where carbapenem resistance rates now approach 37.0% to 50.0% for primary Gram-negative pathogens.

Several clinical factors explain this high resistance baseline. Our patient data show that 39.0% of the cohort had a documented history of antibiotic usage within the preceding three months, creating a strong selection pressure for MDR strains before ICU admission. Other contributing factors include a history of severe infections and the widespread, inappropriate use of broad-spectrum antibiotics in primary care.

As a major tertiary care center, our facility treats a large volume of critically ill patients referred with advanced sepsis and septic shock, who often carry pre-selected, resistant flora. Alarmingly, we identified several pan-drug-resistant (PDR) isolates that were resistant to all tested antimicrobials. The emergence of PDR organisms represents a serious medical challenge, as it leaves clinicians with virtually no viable therapeutic options.

These findings highlight the urgent need for every ICU to develop and regularly update its own local antibiogram. Creating localized antibiotic policies based on regional hospital data can improve the empirical treatment of community-acquired infections, preventing clinical worsening and reducing avoidable ICU admissions. Furthermore, restricting the prolonged or inappropriate use of reserve antibiotics can reduce selective pressure and help restore the efficacy of these drugs over time. Ultimately, understanding local bacterial distribution and susceptibility trends is an essential step toward safer, more effective prescribing patterns.

Limitations of the study

This study has several limitations that should be considered when interpreting the results: (1) single-center design: data were gathered from a single institution, which limits its immediate generalizability; (2) sample size: the small cohort size prevents advanced statistical sub-analyses; (3) ​​​​​​​analytical scope: the study did not include a formal analysis of clinical risk factors, molecular resistance testing, or correlations with patient mortality rates; and (4) environmental factors: institutional infection rates are highly dependent on local infection control practices, specific antibiotic prescribing cultures, and the hospital environment. These institutional variables mean our findings may not fully apply to other hospital or intensive care settings.

## Conclusions

This study underscores the profound challenges of managing antimicrobial resistance in a tertiary care ICU, particularly among a highly vulnerable patient cohort characterized by advanced age, prevalent comorbidities, and significant prior antibiotic exposure. Our findings reveal an overwhelming predominance of GNB, specifically *Klebsiella* spp., *Pseudomonas aeruginosa*, and *Acinetobacter baumannii*, in lower respiratory tract specimens, where diagnostic yields from ET aspirates and BAL fluid were exceptionally high. Crucially, the study demonstrates that traditional empiric therapeutic mainstays, including second- and third-generation cephalosporins, fluoroquinolones, and aminopenicillins, have been largely compromised by extensive resistance. Alarmingly, high resistance rates to piperacillin-tazobactam and a visible escalation in CRE narrow the remaining therapeutic window, leaving reserve agents like colistin and tigecycline as the few viable treatment choices. The identification of PDR strains further signals a critical clinical threshold, threatening complete therapeutic failure in the most severely ill patients.

Ultimately, these data highlight that a generalized antibiotic strategy is no longer sufficient for complex critical care environments. Preserving the efficacy of remaining antimicrobials and improving patient outcomes requires the institutionalization of periodic, ICU-specific antibiograms to guide tailored empirical prescription patterns. Coupled with strict local antibiotic stewardship policies and robust infection control protocols, these localized insights are vital to mitigate the selection pressure driving AMR and to combat the emergence of untreatable superbugs.
